# Acellular foreskin dermal matrix is efficient in supporting the growth of urothelial cells derived from hypospadias patients

**DOI:** 10.3389/fped.2025.1628435

**Published:** 2025-08-04

**Authors:** Zhiqing Cao, Zhenwei Yang, Qunfang Ye, Juntan Xiong, Aozhou Zhu

**Affiliations:** ^1^ Department of Pediatric Surgery, Jiangmen Maternity and Child Health Care Hospital, Jiangmen, Guangdong, China; ^2^ Department of Urology, Shanghai Eighth People’s Hospital, Shanghai, China

**Keywords:** hypospadias, acellular dermal matrix (ADM), urothelium-derived cells, alternative material for tissue engineering, urethral reconstruction

## Abstract

**Introduction:**

Hypospadias is a common congenital defect in males, with surgery remaining the primary treatment option. However, urethral reconstruction procedures often require additional tissue transplantation, which is limited by the availability of suitable tissue sources.

**Methods:**

In this study, we prepared acellular dermal matrix (ADM) from foreskin obtained through circumcision and isolated urothelium-derived cells from patients with hypospadias. We then evaluated the growth of these urothelium-derived cells on the ADM.

**Results:**

Our results confirmed successful decellularization of the foreskin dermal tissues and demonstrated that the resulting ADM exhibited minimal cytotoxicity toward primary urothelium-derived cells. CFSE and CCK-8 staining assays revealed robust urothelial cell growth on the ADM. Furthermore, the ADM with growing urothelium-derived cells displayed superior biomechanical properties compared to native ADM, suggesting that foreskin ADM is an excellent scaffold for urothelial cell growth.

**Discussion:**

These findings indicate that foreskin ADM is a promising alternative material for tissue engineering in the treatment of conditions like hypospadias that require urethral reconstruction.

## Introduction

Hypospadias is a congenital malformation of the male external genitalia, characterized by incomplete fusion of the urethral folds, which leads to the abnormal positioning of the urethral opening (1). It is one of the most common male birth defects, with a global prevalence of 20.9 per 10,000 births between 1980 and 2010, showing a geographical variation (2). While the exact cause of hypospadias remains unclear, several risk factors have been associated with its occurrence, including genetic mutations, insufficient placental nutrition, advanced maternal age, maternal exposure to chemicals and pollutants, and more (1, 3). The primary treatment for hypospadias is surgical intervention, typically recommended between 6 and 18 months of age to reduce psychological stress and behavioral issues (4).

Repairing severe cases of hypospadias often requires the transplantation of additional tissue to reconstruct the urethra. However, the availability of suitable grafts is frequently limited. Although autologous tissues such as genital skin and oral mucosa are commonly used for grafting, the scarcity of these tissues can hinder treatment efficacy (5). As a result, identifying more reliable tissue sources is essential for improving urethral reconstruction in hypospadias patients.

Tissue engineering, a key field within regenerative medicine, applies principles of engineering and life sciences to develop biologically functional substitutes for repairing tissue and organ defects (6). In the context of severe hypospadias, tissue engineering holds therapeutic potential, with a critical need for biocompatible scaffolds that support the growth and expansion of urothelium-derived cells for successful urethral tissue reconstruction (6).

Among various biomaterial scaffolds, acellular dermal matrix (ADM) offers several advantages, including a lower risk of necrosis, lack of immunogenicity, and ease of manipulation (7, 8). These decellularized tissues retain structural proteins such as collagen and proteoglycans, which help preserve their structural integrity while maintaining bioactive growth factors that promote the growth of implanted cells like endothelial and smooth muscle cells (9). Clinical studies on tissue-engineered urethral replacements using human ADM-derived implants have demonstrated favorable outcomes, highlighting the scaffold's excellent biocompatibility (5). However, the limited availability of human skin restricts its wider clinical use.

Foreskin, the loose fold of skin covering the head of the penis, is routinely removed during circumcision, a common procedure globally, particularly among white males (10). Although a small portion of circumcised foreskin is used for research purposes, most of it is discarded as medical waste. Given the high number of circumcisions performed, foreskin could be a reliable source of human ADM. In fact, foreskin-derived ADM has shown potential in promoting skin regeneration during wound healing (11). However, its ability to support urothelial cell growth and its effectiveness in tissue engineering for hypospadias treatment remains largely unexplored.

In this study, we initially collected excised human foreskin and prepared ADM. We then isolated and characterized primary urothelium-derived cells from hypospadias patients. Finally, we assessed the growth of these cells on the foreskin ADM.

## Materials and methods

### Preparation of foreskin ADM

Inflammation-free foreskin samples were collected from subjects who underwent circumcision, with their written consent. The foreskin was thoroughly disinfected with iodine, rinsed repeatedly with saline, and treated with a penicillin and streptomycin solution (4 × 10⁶ IU/L) for 10 min, followed by treatment with chloramphenicol (25 mg/L) for 5 min. The samples were then washed with PBS and placed in 1 mol/L NaCl, shaken at 200 rpm at 37°C for 24 h. Afterward, the epidermis was removed, and the dermis was treated with 0.5% sodium dodecyl sulfate (SDS) at room temperature for 2 h to remove cells. The decellularized dermal matrix was rinsed several times with PBS and then placed in PBS at 4°C for 48 h to eliminate residual SDS. Finally, the ADM was rinsed with PBS and carefully trimmed into a rectangular shape using scissors. Non-decellularized foreskin dermis was used as a control for subsequent histological analyses.

### Histological analysis

Both the non-decellularized foreskin dermis and ADM were fixed in 4% PFA overnight at 4°C. The samples were then paraffin-embedded, sectioned, de-waxed, and rehydrated. Hematoxylin and eosin (H&E) staining and Masson's trichrome staining were performed using kits from Pinuofei Biological (S191003 for H&E, S191006 for Masson's trichrome), following the manufacturer's instructions. The results were captured using a Nikon ECLIPSE-Ci microscope equipped with a digital camera.

### Isolation and culture of primary urothelium-derived cells from hypospadias patients

With parental consent, urethral mucosa tissues were collected from children undergoing repair surgery for perineal hypospadias. The dissected tissues (∼0.5 cm × 0.5 cm) were immersed in DPBS containing penicillin/streptomycin (100,000 U/L) and amphotericin B (1 mg/L) and transported on ice to the laboratory. The tissues were then incubated with 2.24 U/ml Dispase II (Roche) overnight at 4°C. The following day, the mucosal layer was separated from the submucosal layer with forceps, cut into small pieces, and digested with 0.25% trypsin-0.02% EDTA at 37°C for 5 min. The tissues were then placed in DPBS, gently pipetted to form a cell suspension, and passed through a 100-mesh cell filter. The cell suspension was centrifuged at 1,000 rpm for 5 min, and the pellet was resuspended in DMEM/F-12 (1:1, Hyclone). All experiments were approved by the Ethics Committee of Jiangmen Maternity and Child Health Care Hospital ([2022]056).

For cell culture, the cells were seeded into laminin-coated 24-well plates at a density of 5 × 10⁴ cells/ml and cultured in DMEM + 10% FBS at 37°C with 5% CO₂. After 24 h, the culture medium was refreshed, and the medium was changed every 3 days. Once the cells reached 80%–90% confluence, they were passaged using 0.25% trypsin-0.02% EDTA at the same density.

### MTT assay

MTT assay was performed to assess cell viability. Cells were seeded into 96-well plates at a density of 1 × 10⁴ cells per well (200 μl) and cultured for 48 h. Afterward, 10 μl MTT working solution (5 mg/ml, Yeasen) was added to each well, and the plates were incubated at 37°C for 4 h. Then, 150 μl DMSO was added to each well, followed by 10 min of agitation at 37°C. Finally, OD_490_ values were measured using a microplate reader. The assays were performed in triplicate and repeated independently three times.

### Immunofluorescence (IF)

2 × 10⁴ cells were seeded onto each coverslip, which was placed in a 24-well plate and cultured overnight. The cells were then rinsed with PBS and fixed with 4% PFA for 30 min at 4°C, followed by another PBS wash. Cells were permeabilized with 0.5% Triton X-100/PBS for 10 min, washed with PBS three times, and blocked with 2% BSA for 1 h at room temperature. After blocking, the cells were incubated with primary antibody solutions overnight at 4°C. The following day, the cells were washed with PBS, incubated with secondary antibodies for 2 h at room temperature, washed with PBS, counterstained with DAPI, mounted, and imaged using a Nikon DS-Ri2 microscope. The antibodies used were Rabbit anti-CD31 (11265-1-AP, Proteintech, 1:200), Rabbit anti-Cytokeratin 14 (CK14, 10143-1-AP, Proteintech, 1:200), and Goat anti-Rabbit IgG (H + L) Alexa Fluor 488 (SA00006-2, Proteintech, 1:500).

### Carboxyfluorescein succinimidyl ester (CFSE) labeling

2 × 10^4^ cells were seeded onto each coverslip, placed into a 24-well plate, and cultured overnight. The cells were then incubated with 1:1000 diluted CFSE/Hoechst solution for 4 h at 37°C, followed by PBS washing and imaging using a Leica confocal microscope.

### CCK-8 staining assay

5,000 cells were seeded into each well of a 96-well plate containing trimmed ADM and cultured until the indicated time points. At the time of assay, 10 μl CCK-8 solution was added to each well and incubated for 3 h at 37°C. The medium was transferred to another 96-well plate, and OD_450_ values were measured using a microplate reader. The assays were performed in triplicate.

### Biomechanical property assay

To assess the mechanical properties of foreskin ADM, urothelial-cell-seeded ADM, and urothelial cell sheets, samples were trimmed into strips (20 × 50 mm) and clamped onto the sample holders of an STB-1400 Cell Stretching System (Strex Cell) at a speed of 10 mm/min until tearing occurred. Maximum tensile strength and pull forces were recorded and quantified. The experiment was repeated three times independently.

### Xenograft model

To evaluate the host response to implanted ADM constructs, Male nude mice weighing 22–25 g were obtained from the Animal Experiment Center of Xiamen University and housed under standard conditions with controlled temperature (20–24°C), humidity (40%–60%), and a 12-h light/dark cycle, with free access to food and water. For the implantation experiments, sterile 8 × 8 mm ADM samples were surgically implanted into dorsal subcutaneous pockets of C57BL/6 mice, with three mice per experimental group. Following implantation, tissue samples were collected weekly and subjected to histological processing for H&E staining to assess inflammatory responses, ELISA for quantification of IL-6 and TNF-α cytokine levels, and CD31 immunohistochemistry to evaluate vascular ingrowth, while macroscopic observations were performed in parallel to document hair regrowth patterns throughout the study duration.

### Statistical analysis

Statistical analysis and graph generation were conducted using GraphPad Prism (v8.0). One-way ANOVA followed by *post hoc* Tukey's HSD test was used for statistical analysis. Significance was determined at *P < 0.05, **P < 0.01, with ***P < 0.001 and ****P < 0.0001.

## Results

### Characterization of foreskin ADM

**Figure 1:**
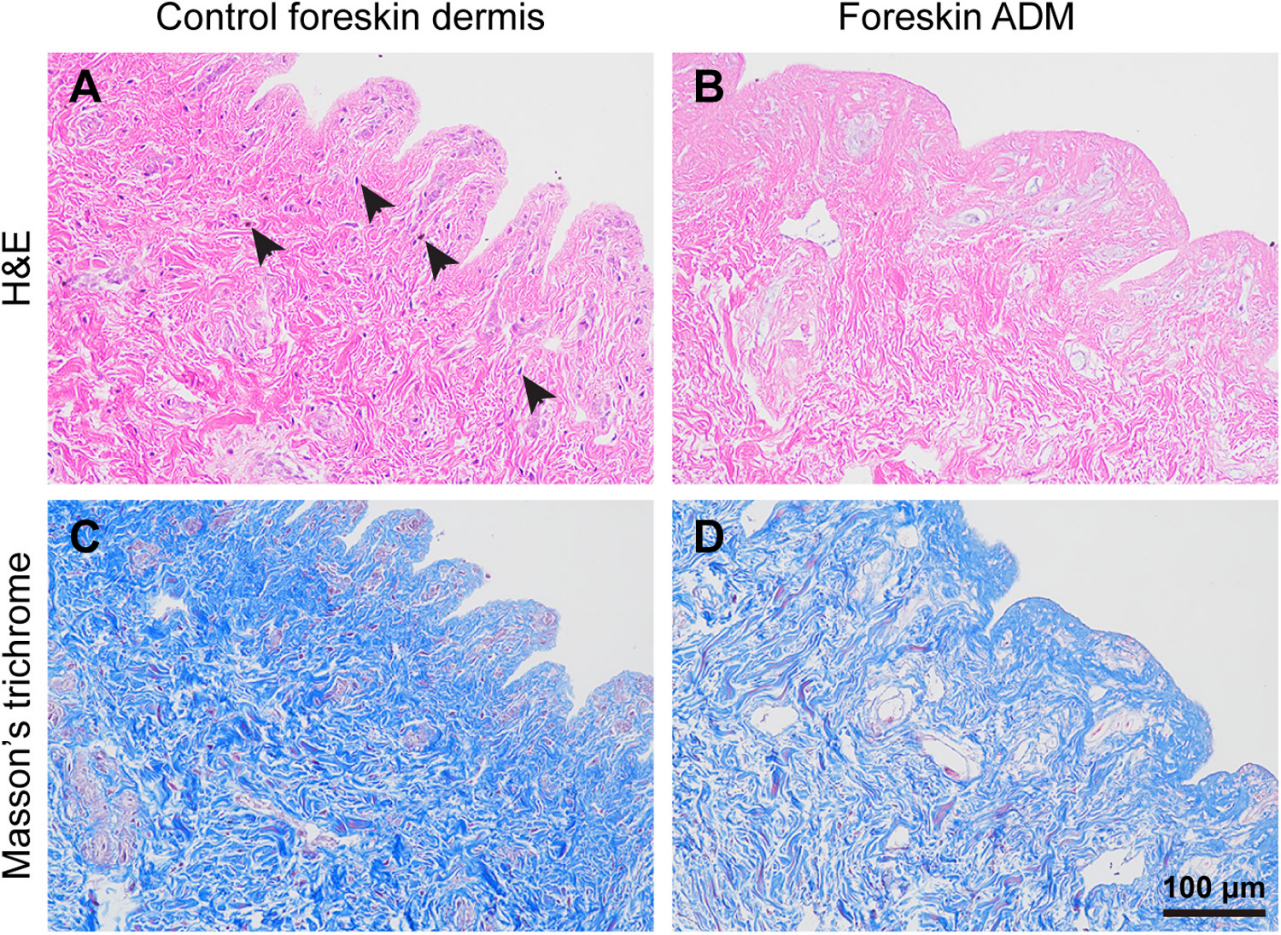
Histology of foreskin dermis and foreskin ADM. **(A,B)** H&E staining illustrating the histological structure of foreskin dermis **(A)** and foreskin ADM **(B) (C,D)** Masson's trichrome staining depicting collagen content and distribution in foreskin dermis **(C)** and foreskin ADM **(D)**.

To assess the efficacy of decellularization, we conducted histological analyses on both untreated foreskin dermis and prepared foreskin ADM. H&E staining revealed that untreated foreskin dermis contained hematoxylin-positive nuclei (indicated by black arrowheads), while foreskin ADM lacked hematoxylin signals, indicating the absence of cellular material (Figures 1A,B). Additionally, to evaluate the collagen content, we performed Masson's trichrome staining. The results demonstrated that although the collagen content (blue staining) in foreskin ADM was slightly reduced, the majority of dermal collagen was retained compared to untreated foreskin dermis (Figures 1C,D). These results confirm the successful preparation of foreskin ADM. Meanwhile, the detection results of hydroxyproline (HYP) content for collagen and glycosaminoglycan (GAG) showed no significant differences between foreskin ADM and control foreskin dermis (Supplementary Figures S1F–G).

### Isolation of primary urothelium-derived cells from hypospadias patients

**Figure 2:**
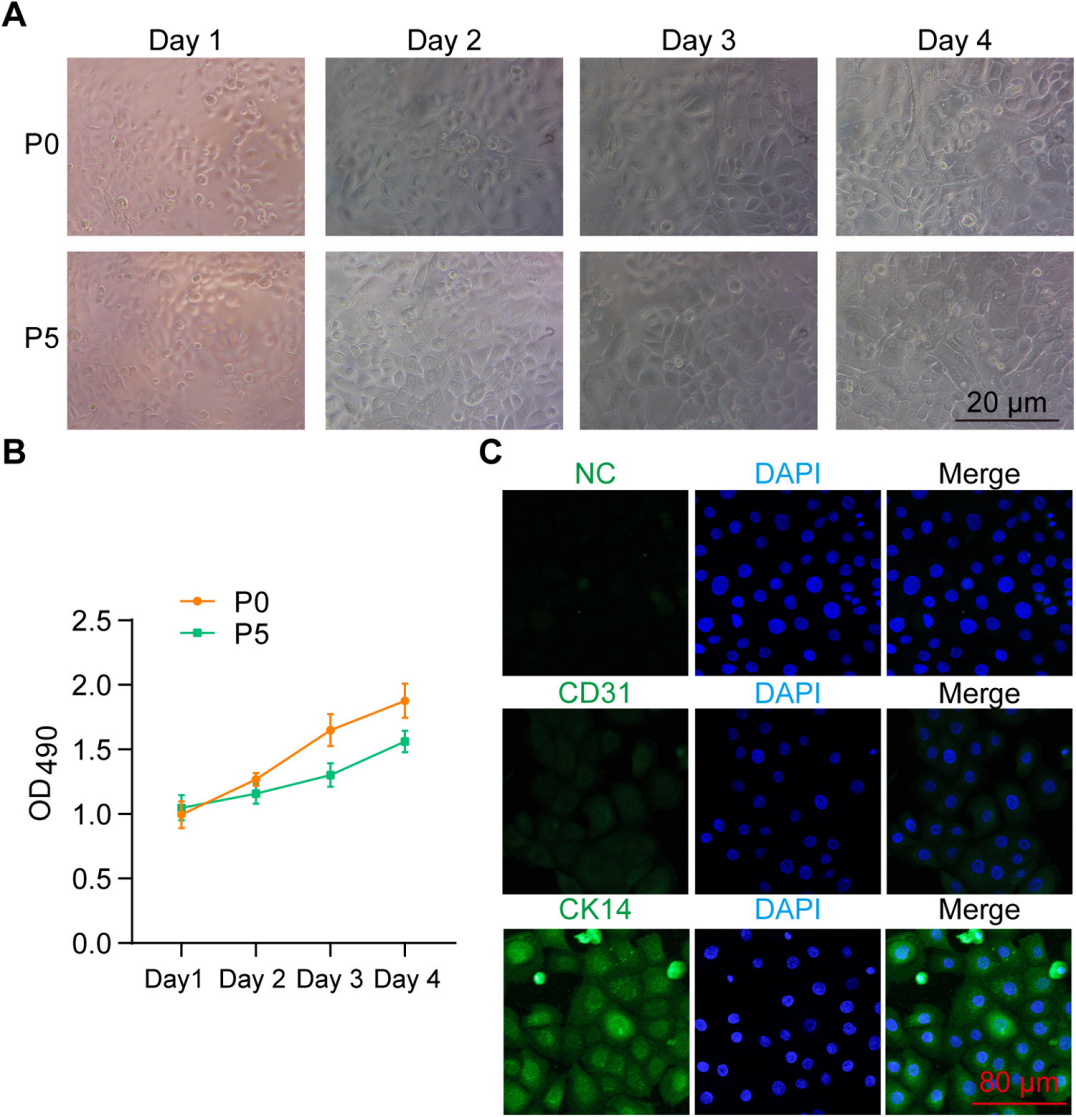
Characterization of primary urothelium-derived cells. **(A)** Bright-field images showing the morphology of isolated primary urothelium-derived cells. P0 and P5 indicate cells at passage 0 and passage 5, respectively. **(B)** MTT assay results demonstrating the viability of P0 and P5 urothelium-derived cells over a 96-h culture period. **(C)** Immunocytochemistry results showing the expression of CD31 and CK14 in the primary urothelium-derived cells. NC stands for negative control.

To characterize the isolated primary urothelium-derived cells, we first monitored their growth under a bright-field microscope over 96 h. The cells exhibited typical urothelial morphology and reached 100% confluence within 48 h after initial inoculation. The growth potential was maintained even after being passaged for five generations (Figure 2A). MTT assays were performed to quantify the viability of these cells, showing that their viability was slightly reduced after five passages (Figure 2B). Furthermore, IF staining revealed that these cells were negative for the endothelial marker CD31 but expressed the epithelial marker CK14 (Figure 2C), confirming their identity as urothelium-derived cells.

### Evaluation of the cytotoxicity of foreskin ADM to primary urothelium-derived cells

**Figure 3:**
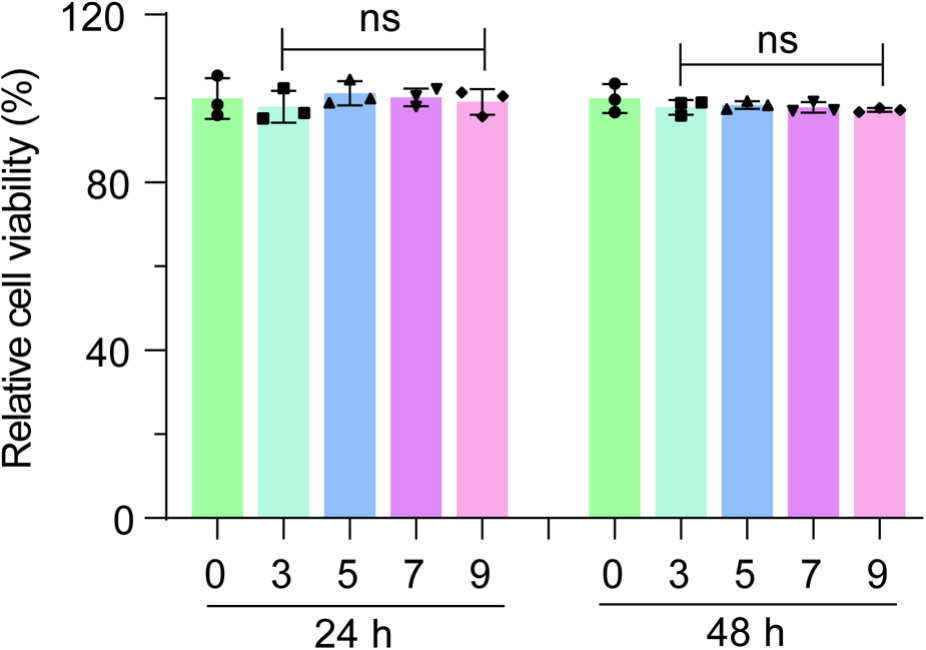
Evaluation of cytotoxicity of foreskin ADM on primary urothelium-derived cells. MTT assay results showing the viability of primary urothelium-derived cells cultured in media conditioned with various concentrations of foreskin ADM soaking solution for 24 or 48 h. One-way ANOVA was used to assess statistical significance. “ns” denotes no significant difference.

To assess any potential adverse effects of foreskin ADM on primary urothelium-derived cells, we soaked the ADM in culture medium to create a conditioned medium, which was then added to the urothelial cell culture. After 24 or 48 h, MTT assays were conducted to measure cell viability. The results indicated that the ADM-conditioned medium had minimal impact on the viability of primary urothelium-derived cells (Figure 3), demonstrating that foreskin ADM is biocompatible and safe for use with urothelium-derived cells.

### Assessment of the growth of primary urothelium-derived cells on foreskin ADM

**Figure 4:**
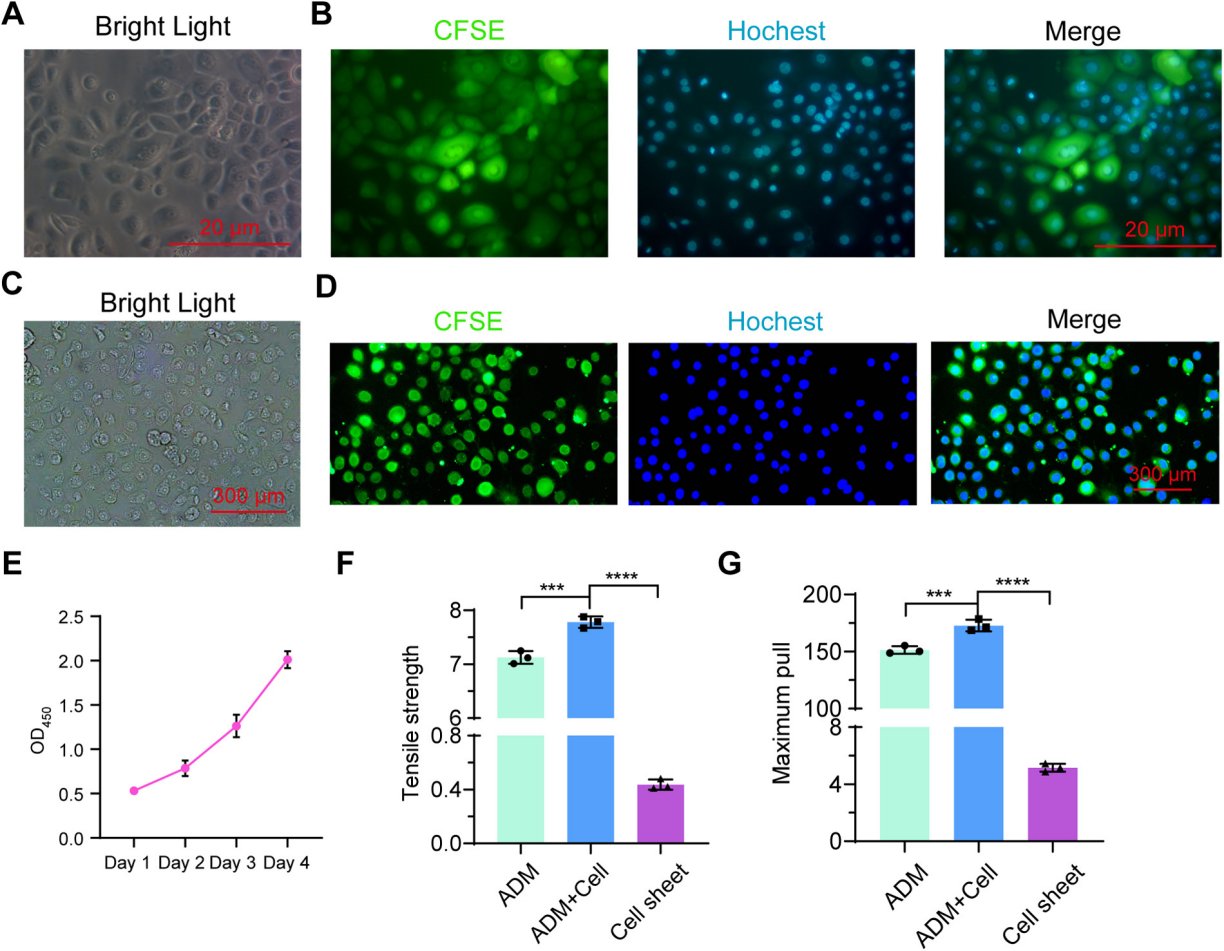
Assessment of primary urothelium-derived cells cultured on foreskin ADM. **(A,B)** Morphology of CFSE-labeled primary urothelium-derived cells under bright field **(A)** and fluorescent field **(B)** microscopy. **(C,D)** Morphology of CFSE-labeled primary urothelium-derived cells cultured on ADM for 4 days under bright field **(C)** and fluorescent field **(D)** microscopy. **(E)** CCK-8 assay results showing the proliferation of urothelium-derived cells growing on ADM over time. **(F,G)** Graphs showing maximum tensile strength **(F)** and pull force **(G)** for ADM, cell-seeded ADM, and cell sheets.

To directly evaluate the growth of primary urothelium-derived cells on foreskin ADM, we labeled the urothelium-derived cells with CFSE (Figures 4A,B). These fluorescence-labeled cells were inoculated onto the foreskin ADM for co-culture. After 96 h, we observed that the majority of labeled cells remained viable (Figures 4C,D), with continued cell division throughout the 4-day culture period, as shown by the CCK-8 assay results (Figure 4E). Furthermore, we examined the biomechanical properties of the ADM, ADM with growing cells, and the urothelial cell sheet. The findings indicated that the maximum tensile strength and pull force of the cell-growing ADM were significantly higher than that of native ADM and urothelial cell sheets (Figures 4F,G). These results suggest that primary urothelium-derived cells can grow robustly on foreskin ADM.

### Assessment of host response to foreskin ADM implantation in a murine model

To systematically assess the host response to ADM implantation, we established three experimental groups: Group 1 (ADM subcutaneous implantation), Group 2 (ADM dorsal subcutaneous implantation with cells), and Control (Sham operation). The ADM scaffolds were surgically implanted into the dorsal subcutaneous space of mice, with tissue samples harvested at 2 weeks post-operation (*n* = 3 per group). Histological evaluation through H&E staining and cytokine analysis via ELISA demonstrated comparable levels of IL-6 and TNF-α expression between experimental and control groups, indicating minimal inflammatory response (Supplementary Figures S1A,D–E). CD31 immunohistochemical analysis demonstrated higher expression levels in experimental groups compared to controls in the study period (Supplementary Figure S1C). Macroscopic examination confirmed the hairless nature of ADM implantation sites, in contrast to the normal hair growth observed in control areas (Supplementary Figure S1B). These collective findings suggest that foreskin-derived ADM exhibits excellent biocompatibility and promotes vascularization while maintaining its critical hairless property for urethral tissue engineering applications.

## Discussion

Although significant progress has been made in the development of synthetic scaffolds for tissue engineering, limitations in their biocompatibility, biodegradability, and mechanical properties have restricted their clinical use. As a result, natural biological scaffolds continue to play a major role in tissue reconstruction, including hypospadias repair. The search for more reliable sources of such scaffolds is crucial to meet the increasing demand for tissue reconstruction. The foreskin-derived acellular dermal matrix (ADM) presents several distinctive advantages as a biomaterial scaffold. Sourced as routine medical waste from neonatal circumcision procedures, this ADM offers an ethically uncomplicated and readily available tissue source. The young donor age contributes to superior biological properties, with preserved levels of critical growth factors including VEGF and FGF, along with essential extracellular matrix components such as laminin and hyaluronic acid, which collectively enhance its regenerative potential. Characterized by an optimal native thickness of approximately 0.3 mm, this ADM is particularly well-suited for mucosal tissue engineering applications including urethral reconstruction, eliminating the need for potentially damaging thinning procedures required for conventional ADM products. Furthermore, the production process demonstrates considerable cost-effectiveness, achieving over 50% reduction in manufacturing costs compared to commercially available cadaveric-derived ADM. These combined attributes position foreskin-derived ADM as a clinically valuable and economically viable scaffold material for regenerative medicine applications.

In this study, we explored the potential of using foreskin ADM to support the growth of primary urothelium-derived cells isolated from hypospadias patients. Our analysis revealed that the decellularization process was efficient, and the resulting ADM retained an intact dermal collagen network. To evaluate the ability of foreskin-derived ADM to support urothelial cell growth, we isolated primary urothelium-derived cells and found that the ADM showed minimal cytotoxicity to these cells. Further investigation confirmed that the urothelium-derived cells thrived on the ADM, suggesting its great therapeutic potential in urethral reconstruction surgery.

ADM from various sources has a broad range of clinical applications, such as burn surgery, breast reconstruction, andrological surgeries, orthopedic procedures, and craniofacial surgery (8). In the context of hairy skin, ADM is useful for repairing small wounds (11) but is less ideal for large wounds due to the lack of appendages such as hair follicles, sweat glands, and pigmentation, which can lead to complications (12). By contrast, the hairless nature of ADM is advantageous for urethral tissue repair, as these tissues are naturally hairless. A significant portion of hypospadias patients who undergo urethral repair suffer from complications caused by hair growth in the urethral tissues (13, 14). However, because decellularization disrupts the dermal matrix microenvironment to some extent (15), it remains uncertain whether this hairless characteristic persists in ADM. Further studies are necessary to evaluate the long-term effects of using foreskin-derived ADM in urethral reconstruction.

For clinical vascularization strategies, we propose three approaches: first, wrapping ADM grafts with vascular pedicle tissues, a technique widely used in urethral reconstruction that can provide immediate blood supply support; second, promoting neovascularization through ADM surface modification technology; and third, combining minimally invasive surgical techniques to establish preliminary vascular networks before implantation. The comprehensive application of these strategies will significantly improve the early vascularization efficiency of ADM grafts, providing important references for subsequent clinical applications. Current animal experimental results have preliminarily verified the pro-angiogenic potential of ADM itself, but comparative studies of different vascularization schemes will be the focus of future large animal experiments.

It is worth noting that despite retaining an intact collagen network, the collagen density in ADM appears to decrease following the decellularization process. This reduction may be due to the harsh chemical treatments used and could potentially be mitigated by modifying the decellularization solution components. Therefore, it is important to investigate alternative decellularization methods, such as enzyme-based protocols (16), to assess the biocompatibility of the resulting ADM with urothelium-derived cells. Furthermore, considering the increasing clinical demand for ADM beyond urethral tissue reconstruction (17), it would be beneficial to assess the cytotoxicity and biocompatibility of foreskin ADM with other cell types, thereby broadening its applications.

It is important to emphasize that while our primary conclusions are derived from *in vitro* assays, they have been partially supported by preliminary *in vivo* experiments. To fully evaluate the potential of foreskin ADM for urethral tissue reconstruction and its long-term repair efficacy, more comprehensive *in vivo* experiments and clinical trials are required. The current findings are primarily based on *in vitro* experiments, though initial animal studies have corroborated some aspects of the pro-angiogenic potential of the ADM. Its complete integration efficacy within the complex *in vivo* microenvironment still requires further verification through large animal experiments. Additionally, the observed reduction in collagen density following the decellularization process suggests the need for future optimization of decellularization protocols.

In summary, our data suggest that foreskin is a potentially reliable source of ADM that can effectively support the growth of primary urothelium-derived cells. This study systematically evaluated the application potential of foreskin-derived acellular dermal matrix (ADM) as a urethral reconstruction scaffold, demonstrating significant clinical translational value. Through optimized decellularization protocols, the obtained ADM not only completely removed cellular components while preserving intact collagen network architecture and crucial extracellular matrix constituents, but also exhibited excellent biocompatibility with primary urothelial cells. This makes it a promising scaffold for urothelial tissue repair, which could aid in the treatment of conditions such as severe hypospadias. Particularly noteworthy are its unique clinical advantages: the natural 0.3 mm thickness makes it ideally suited for direct urethral repair without secondary processing, and its production costs are significantly lower than commercial ADM products, providing a cost-effective solution to address the clinical shortage of urethral repair materials.

## Data Availability

The original contributions presented in the study are included in the article/Supplementary Material, further inquiries can be directed to the corresponding author.

## Supplementary material

Supplementary Figure S1 Mouse model evaluation of foreskin adm host response. **(A,B)** Histological evaluation of foreskin ADM and control samples by H&E staining **(A)** and hair follicle growth assessment **(B)**. **(C)** CD31 immunohistochemical analysis. **(D–E)** Quantitative analysis of inflammatory markers (IL-6/TNF-α) by ELISA. **(F,G)** Biochemical quantification of glycosaminoglycan (GAG) **(F)** and hydroxyproline (HYP) **(G)** content in ADM scaffolds.
