# Supplementary figures and images for: Acellular foreskin dermal matrix is efficient in supporting the growth of urothelial cells derived from hypospadias patients
Source: Front Pediatr. 2025 Aug 4;13:1628435. doi: 10.3389/fped.2025.1628435 (PMC12358349; doi:10.3389/fped.2025.1628435)

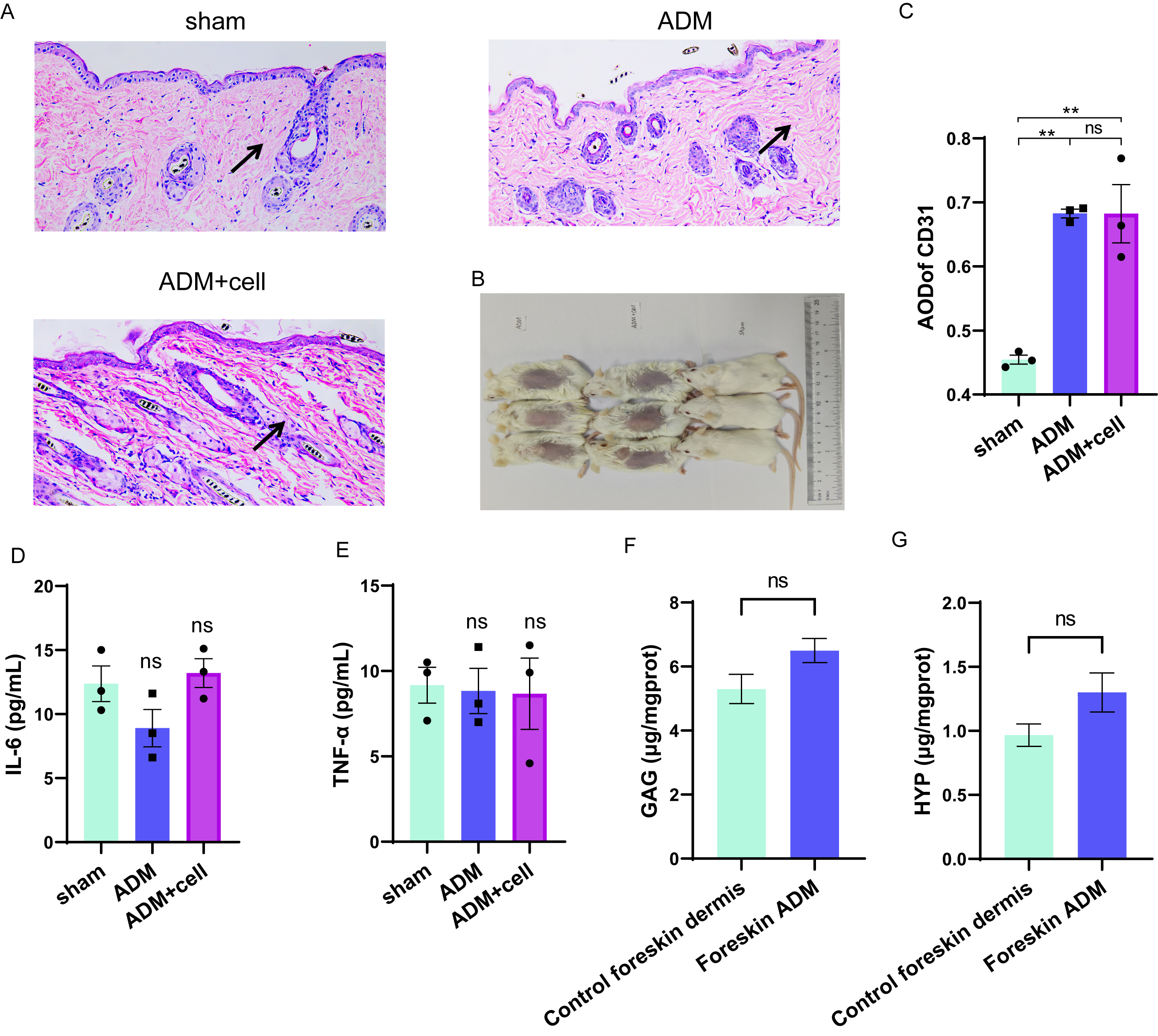

Supplement: Supplementary Figure S1 — Mouse model evaluation of foreskin adm host response. (A,B) Histological evaluation of foreskin ADM and control samples by H&E staining (A) and hair follicle growth assessment (B). (C) CD31 immunohistochemical analysis. (D–E) Quantitative analysis of inflammatory markers (IL-6/TNF-α) by ELISA. (F,G) Biochemical quantification of glycosaminoglycan (GAG) (F) and hydroxyproline (HYP) (G) content in ADM scaffolds. [file Image1.tif]
